# TORNADO: Intermediate Results Orchestration Based Service-Oriented Data Curation Framework for Intelligent Video Big Data Analytics in the Cloud

**DOI:** 10.3390/s20123581

**Published:** 2020-06-24

**Authors:** Aftab Alam, Young-Koo Lee

**Affiliations:** Data and Knowledge Engineering Laboratory, Department of Computer Engineering, College of Electronics and Information, Kyung Hee University, Yongin-si, Gyeonggi-do 17104, Korea; aftab@khu.ac.kr

**Keywords:** video big data curation, distributed video analytics, service-oriented architecture, intermediate results orchestration, big data analytics

## Abstract

In the recent past, the number of surveillance cameras placed in the public has increased significantly, and an enormous amount of visual data is produced at an alarming rate. Resultantly, there is a demand for a distributed system for video analytics. However, a majority of existing research on video analytics focuses on improving video content management and rely on a traditional client/server framework. In this paper, we develop a scalable and flexible framework called TORNADO on top of general-purpose big data technologies for intelligent video big data analytics in the cloud. The proposed framework acquires video streams from device-independent data-sources utilizing distributed streams and file management systems. High-level abstractions are provided to allow the researcher to develop and deploy video analytics algorithms and services in the cloud under the as-a-service paradigm. Furthermore, a unified IR Middleware has been proposed to orchestrate the intermediate results being generated during video big data analytics in the cloud. We report results demonstrating the performance of the proposed framework and the viability of its usage in terms of better scalability, less fault-tolerance, and better performance.

## 1. Introduction

Videos are recorded and uploaded to the cloud regularly. Sources that are actively contributing to video generation include CCTV, smartphones, drones, and many more, which have resulted in a big data revolution in video management systems. Various leading industrial organizations have successfully deployed video management and analytics platforms that provide more bandwidth and high-resolution cameras collecting videos at scale and has become one of the latest trends in the video surveillance industry. For example, YouTube users upload more than 400 h of videos per minute [1], and more than one hundred and seventy million video surveillance cameras have been installed in China only [2]. It has been reported that the data generated by Internet of things (IoT) devices will see a growth rate of 28.7% over the period 2018–2025, where surveillance videos are the majority shareholder, i.e., 65% [3]. Such an enormous video data is considered as “video big data” because a variety of sources generate a large volume of video data at high velocity that holds high value. Video data are acquired directly from real-world domains and meet the veracity characteristic. Handling large-scale complex video data is not worthwhile utilizing conventional data analysis approaches. Video big data pose challenges for video management, processing, mining, and manipulation. Therefore, more comprehensive and sophisticated solutions are required to manage and analyze large-scale unstructured video data. Furthermore, due to the large volume of video data, it requires a flexible solution to store and mine for possible decision-making. However, large-scale Intelligent Video Analytics (IVA) becomes a reality on the rise of big data analytics and cloud computing technologies.

Big data technologies, such as Hadoop [4] or Spark [5] ecosystem, are software platforms designed for distributed computing to process, analyze, and extract the valuable insights from large datasets in a scalable and reliable way. Cloud computing is a model for enabling ubiquitous, convenient, and on-demand network access to a shared pool of configurable computing resources that can be rapidly provisioned and released with minimal management effort or service provider interaction [6]. The cloud is preferably appropriate to offer the big data computation power required for the processing of these large datasets. For big data analytics, numerous cloud platforms have been developed, including IBM Big Data Analytics [7], Amazon web service [8], and many more.

Recently, the deployment of distributed computing technologies in the cloud for video big data analytics has been the center of attraction in academics and industry. In the literature, some efforts have been made to propose a Cloud-based Video Analytics System (CVAS) for online and offline IVA, for example, [9,10,11]. The focus of these studies is more on video analytics (value extraction) and overlook the data curation issues encompassing value, volume, velocity, and variety management throughout the lifecycle of IVA in the cloud. Data curation is the active management of data over its life cycle, from creation, acquisition, and initial storage to the time when it is archived or becomes obsolete [12]. Industrial CVAS, such as Checkvideo [13], Intelli-Vision [14], assist consumers with limited functionalities, i.e., real-time IVA service subscription. Google released a cloud-based video intelligence API to generate video metadata [15] and provided it as a black box to the developer. It does not allow the developer/researcher to plug new IVA algorithms or to extend its functionality. Likewise, the understanding, configuration, and operationalization of big data technologies for IVA in the cloud are tedious and time-consuming, especially in an educational environment. Furthermore, the IVA lifecycle in the cloud spin around IVA approaches. The existing solutions also do not consider factors like the management of high-level and low-level features while deploying IVA algorithms. Motivated by these limitations in existing work, we propose and implement a comprehensive intermediate results orchestration based service-oriented data curation framework for large-scale online and offline IVA in the cloud known as TORNADO.

The contribution of TORNADO is many folds, but principally, it can be aligned with the deployment of IVA algorithms and services in the cloud for video big data analytics. The main contributions of the proposed TORNADO are listed below.

- We propose a distributed data curation framework for video big data analytics in the cloud and encompasses key components like role-based access controller, device-independent video stream acquisition and synchronization, lifelong video stream monitoring tool, and big data persistence.
- High-level abstraction on top of big data technologies have been developed and optimized for video big data analytics to hide the complexity of big data stack.
- The proposed TORNADO effectively resolves the data curation issues throughout the life cycle of the IVA pipeline by developing distributed data management modules both for real-time and offline analytics.
- TORNADO provides IVA algorithms and service creation and publishing APIs that enable developers and researchers to author and publish contextual and domain-specific IVA algorithms and services, which are made available to the developers while following as-a-Service (aaS) model [16], i.e., IVA-Algorithm-as-a-Service (IVAAaaS) [17] and IVA-as-a-Service (IVAaaS) [18]. Under the Customer-to-Customer (C2C) business model [19], the IVAAaas bridge the gap between the IVA algorithm creator and IVA service developer.
- We propose and optimize a unified scale-out middleware called IR Middleware against issues like big dimensionality, intermediate results, and IVA pipeline orchestration.
- We implement the proposed framework and conduct extensive experiments to validate our claims.

The rest of the paper is planned as: Section 2 provides the related work; background and nomenclature are discussed in Section 3. Section 4 thoroughly explains the proposed framework. Section 5 presents the execution scenarios. Evaluation has been presented in Section 6 and comparison with state-of-the-art solutions can be found in Section 7. Finally, the conclusion is written in Section 8.

## 2. Related Work

Real-time video surveillance and unstructured batch video data analytics in the cloud is an emerging research domain, and has attracted the attention of researches and practitioners. The state-of-the-art literature is proof of the fact that there is an increasing interest in adopting big data technologies for video analytics in the cloud.

In the context of CVAS, Hossain [20] pointed out some significant design considerations and proposed a cloud-based multimedia framework. The proposed framework consists of core components like service directory, cloud manager, monitoring and metering, resource allocation manager, heterogeneous contents manager, consumer manager, and service stack. A prototype was developed of the proposed system and documented the workload of video analytics services and storage tasks. The design considerations of this architecture are beneficial for the research community but are unable to address issues like scalability, fault-tolerance, and video analytics plugins. This architecture also does not support distributed video analytics in the cloud, IVA life cycle, the features management. Ajiboye, S.O. et al. [9] stated that the network video recorder is already equipped with intelligent video processing capabilities but complained about its limitations, i.e., isolation, and scalability. To resolve such issues, they proposed a general high-level theoretical architecture called Fused Video Surveillance Architecture. The design goals were cost reduction, unify data mining, public safety, and intelligent video analytics. The proposed architecture consists of four layers, i.e., application layer (responsible for system administration and user management), services layer (for storage and analytics), network layer, and physical layer (physical devices like camera, etc.). They guaranteed the compatibility of their proposed architecture with the hierarchical structure of computer networks and emerging technologies. However, this is just a conceptual model for surveillance systems and does not give explicit architectural details. Liu, X. et al. [10] came out with a cloud platform for large-scale video analytics and management. They stated that the existing work failed to design a versatile video management platform in a user-friendly way and to use Hadoop to tune the performance of video processing effectively. They develop a cloud platform while using big data technologies, i.e., Hadoop and MapReduce. This architecture only resolves the volume issue related to large-scale video management in the cloud and cannot be considered as a candidate solution for CVAS.

Zhang et al. [21,22] stated that the historical video data could be used with the updated video stream in order to know the status of an activity, for example, identifying the current situation of traffic on the road, and to predict future activity. To make it possible, they proposed a video cloud-based service-oriented layered architecture called Depth Awareness Framework. The proposed framework consists of four layers, i.e., data retrieval layer, offline video analytics layer, online video processing layer, and domain service layer. The data service layer is supposed to handle large-scale video data and webcam streams. The offline layer is used to operate on the batch videos, whereas online processing takes place in a real-time video processing layer. On the top of the proposed cloud platform, they implemented a deep convolution neural network for obtaining in-depth raw context data inside a big video, and a deep belief network-based method to predict workload status of different cloud nodes, as a part of knowledge on the system running status. In another study, Zhang et al. [11] proposed a cloud-based architecture for large-scale intelligent video analytics called BiF. BiF architecture considered non-functional architectural properties and constraints, i.e., usability, scalability, reliability, fault tolerance, data immutability, recomputation, storing large objects, batch processing capabilities, streaming data access, simplicity and consistency. The BiF architecture consists of four main layers, i.e., data collection layer, batch layer, real-time layer, and serving layer. The data collection layer collects the streaming video frames from the input video sources (camera). The data curation layer forwards the video frames to the batch layer and streaming layer for batch processing and real-time analytics respectively. The service layer is to query both views (batch and real-time views) and integrate them to answer queries from a client. These two frameworks combine real-time and offline video analytics, but it has some limitations. These frameworks are lacking the details of data acquisition, architectural details, and there are no technical details on how to develop new video analytics plugins and to manage the intermediate results.

In literature, there are some more studies in which efforts are made to resolve CVAS related issues. Pereira, R. et al. [23] was motivated by the fact that digital video compression is crucial for storage in the cloud and transmission. In this context, they proposed a cloud-based distributed architecture for video compression based on the Split-Merge technique while using the MapReduce framework. Liu et al. [24] used distributed technologies, i.e., Hadoop and MapReduce, for video sharing and transcoding purposes. Likewise, Lin, C.-F. et al. [25] implemented a prototype of a cloud-based video recorder system. A CVAS was proposed by Ananthanarayanan et al. [26,27] called Killer App to meet the real-time demands of video analytics and to address latency, bandwidth, and provisioning challenges. Furthermore, various leading industrial CVAS have successfully been deployed, and they allow the consumers a service subscription. Some organizations provide real-time video analytics for security, while others to extract metadata from video contents with the aim of indexing and searching. Some of the popular real-time video stream analytics service providers are Check Video [13], Eagle Eye Networks [28], and Intelli-Version [14]. These companies provide cloud-based real-time video analytics services. However, they did not offer video analytics APIs aaS to the developers and researchers to build and deploy new video analytics algorithms and services. Recently, Google released a cloud-based video intelligence API to generate video metadata [15] and provided it as a black box to the developer. These systems are developed for commercial use and do not allow developers and scientists to create new video data mining algorithms and services.

Unlike all of the above approaches, TORNADO is an attempt to fill the research gap by proposing, implementing, and evaluating a novel cloud-based framework for video big data analytics. TORNADO is designed on top of state-of-the-art distributed computing and data management technologies. The proposed framework is composed of different types of components and APIs, which can be deployed on various types of computing clusters independently with the intentions of scalability, fault-tolerance, and extensibility. Efforts have been made to provide high-level abstractions to hide the complexity of the cloud and distributed computing technologies. These high-level APIs assist researches in focusing on distributed IVA logic instead of focusing on the platform. In this direction, the video data acquisition APIs allow external real-time video data sources to register and collect the batch video data from the registered users. The acquired data are then maintained securely through a distributed messaging system and a distributed file system. TORNADO provides role-based access to the video data through APIs that make the life of the developer easy while developing new video data mining algorithms and services. IVA experts can develop and register contextual distributed IVA APIs, which is then available aaS to the users. TORNADO assists the IVA life cycle by managing the high-level and low-level features through Intermediate Results (IR) Middleware during the IVAs pipelining. Furthermore, TORNADO develops anomaly detection on the video streaming instances based upon the contextual IVA algorithm.

## 3. Background and Nomenclatures

**Figure 1:**
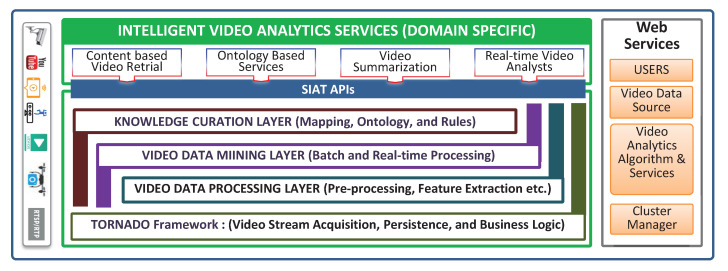
SIAT (ongoing collaborative research project) architecture.

TORNADO is the foundation layer of an ongoing collaborative research project called SIAT [29,30]. SIAT is a layered architecture for distributed IVA in the cloud, as shown in Figure 1. TORNADO is the base layer that allows the other layers to develop IVA algorithms and services. The Video Data Processing Layer (VDPL) is in charge of pre-processing and extracting the significant features from the raw videos and input to the Video Data Mining Layer (VDML). The VDML is accountable for producing the high-level semantic result from the features generated by the VDPL. The Knowledge Curation Layer (KCL) [31] deploys video ontology and creates knowledge based on the extracted higher-level features obtained from VDML. The VDPL and VDML can use the TORNADO base layer under the aaS paradigm. The VDPL, VDML, and KCL are pipelined in a specific context and become an IVA service to which TORNADO users can subscribe video data sources under the IVAaaS paradigm. However, the scope of this paper is the TORNADO layer only.

**Figure 2:**
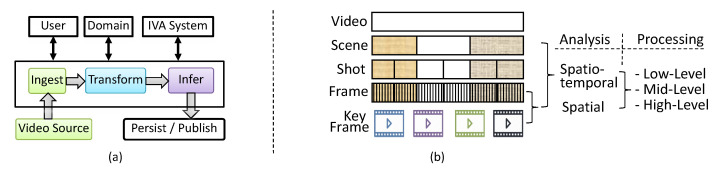
(**a**) A generic Intelligent Video Analytics (IVA) pipeline. (**b**) Hierarchical representation of video units.

The process of IVA undergoes different phases, as shown in Figure 2a. The Video Source are the sources that either generate video streams from sources connected directly to real-world domains such as IP-camera or can be already acquired videos in the form of datasets. IVA in the cloud are performed either on the video streams or on video datasets and is referred as Real-time IVA (RIVA) and Batch IVA (BIVA), respectively. In the context of IVA, a video can be represented in a hierarchy, as shown in Figure 2b. A given video may be decomposed into its constituent units either in the temporal or spatial domain. We call mini-batch to a group of frames with respect to time that belongs to a Video Source. The size of the mini-batch is dependent on the contextual IVA analysis. Such constituent units are further subject to low, mid, or/and high-level processing. In the low-level processing, primitive operations are performed, for example, noise reduction and histogram equalizer, where the input and output are a sequence of frames. The mid-level processing extracts features from the sequence of frames, for example, segmentation, description, classification. The high-level processing makes sense of an ensemble of recognized objects, and perform the cognitive functions normally associated with vision.

The input and output of an IVA algorithm can be a sequence of frames or features. We call these features IR. Multiple algorithms are pipelined to build a domain-specific IVA service. The input and output of an IVA service is restricted to Video Source and IR, respectively. Once an IVA service is developed and deployed then TORNADO users can subscribe a Video Source under the IVAaaS paradigm. The User represents the stakeholder of the proposed framework, i.e., such as administrator, the consumer, and researchers/practitioners. A Domain. A Domain is a specific real-word environment, for example, street, shop, road traffic, for which an IVA service needs to be built. The combination of IVA service and hardware constitutes an IVA System where IVA solutions can scale-out and can run fast.

## 4. Proposed TORNADO Framework

**Figure 3:**
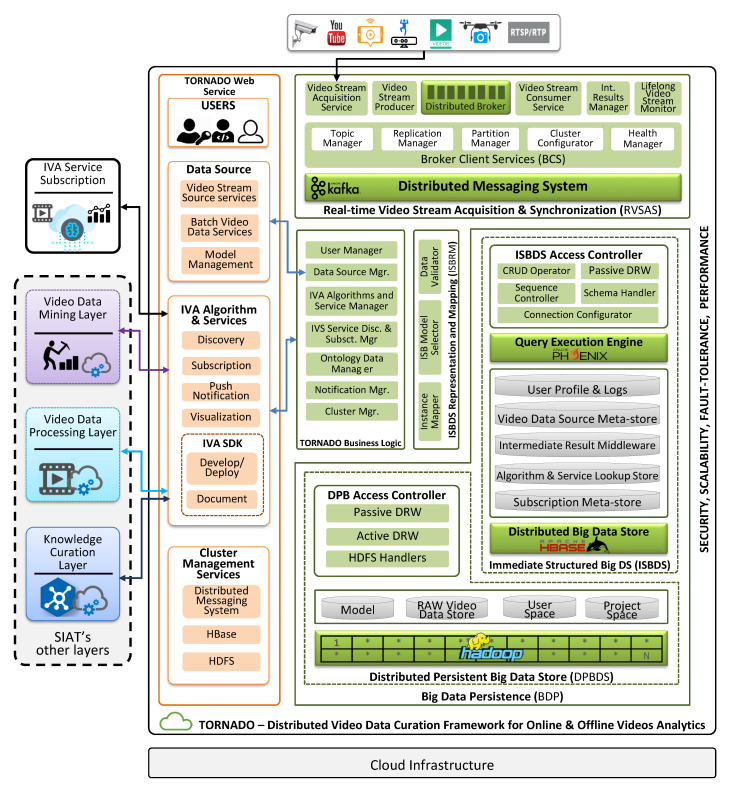
Proposed TORNADO framework architecture.

Formally, we describe the main components of TORNADO in this section and the technical details in the next subsections. As illustrated in Figure 3, TORNADO is composed of six components, i.e., Real-time Video Stream Acquisition and Synchronization (RVSAS), Immediate Structured Big Data Store (ISBDS), Distributed Persistent Big Data Storage (DPBDS), ISBDS Representation and Mapping (ISBRM), TORNADO Business Logic, and TORNADO Web Services.

The RVSAS component provides interfaces and acquires large-scale video streams from device-independent video stream sources for on-the-fly processing. The video stream sources are synchronized based upon the user identification and the timestamp of the video stream generation. Then, it is queued in the form of mini-batches in distributed stream buffer for RIVA. The RIVA may vary in the context of a business domain, cross-linked with the video stream sources and user’s profile. RIVA services are deployed in a cluster of computers for distributed video stream processing to extract the value for contextual decision making. The video stream queued in the form of a mini-batch can be accessed while using Video Stream Consumer Services (VSCS). During RIVA service pipelining, the IR are maintained through Intermediate Results Manager (IR-Manager). Similarly, RVSAS is equipped with a Lifelong Video Stream Monitor (LVSM) to provide a push-based notification response to the client with the help of a publish-subscribe mechanism. The extracted values (features and anomalies) and the actual video streams are then persisted into ISBDS and DPBDS, respectively.

ISBDS component is responsible for storing and managing large-scale structured data in a distributed environment according to the business logic implemented by TORNADO Business Logic. The structured data is related to users, access rights, VDPL, VDML, KCL, metadata of the video data stream sources, batch datasets, big models, and service subscription information. This module also orchestrates the IR and anomalies being generated by a domain-specific IVA service. According to the demands of TORNADO, two types of operations are required to be performed on the ISBDS, i.e., random read-write operation against the real-time queries and bulk load and store operations for offline analytics. For such operations, we develop and deploy ISBDS Access Controller to access the underlying data securely against random and scan read-write operations.

DPBDS is built on top of Hadoop Distributed File System (HDFS) and is responsible for providing permanent and distributed big data persistence to the raw video data, big models, and also supposed to maintain the actual IVA plugins deployed by other layers. During the contextual distributed offline video analytics, batch video data and models are needed to be loaded. Similarly, different HDFS file operations are required, for example, access permission, file creation. In this context, we exploit the services of DPBDS Access Controller.

The ISBRM component is responsible for validating and mapping the contextual data to the respective data stores according to the business logic of TORNADO. TORNADO Business Logic hides the complexity of the proposed system by establishing a well-defined set of uniform and consistent operations. TORNADO is built to provide IVAAaaS and IVAaaS over the web. Thus, we develop TORNADO Web Services on top of TORNADO Business Logic to allow the users to utilize the functionality of the proposed framework over the web. Further technical details of each component are described in the following subsections.

### 4.1. Real-Time Video Stream Acquisition and Synchronization

Handling a tremendous amount of video streams, both processing and storage are subject to loss [11]. To handle a large-scale video stream acquisition in real-time and to ensure scalability and fault-tolerance, we develop the RVSAS component while exploiting a distributed message-oriented middleware known as Apache Kafka [32]. The RVSAS component is responsible for handling and collecting real-time video streams and is composed of six modules, i.e., Broker Client Services (BCS), Video Stream Acquisition Service (VSAS), Video Stream Producer (VSP), VSCS, IR-Manager and LVSM.

#### 4.1.1. Broker Client Services

We utilize Apache Kafka to collect video streams in the form of mini-batches from the producer cluster, buffer in the Kafka Broker, and then route mini-batches to the consumer’s cluster. To manage the Kafka Broker for large-scale real-time video stream management and according to the business logic of the proposed Framework, we develop a module for this purpose known as BCS. The BCS is composed of five sub-modules, i.e., Topic Manager, Partition Manager, Replication Manager, Cluster Configurator, and Cluster Health Manager, as shown in Figure 3.

Topic Manager sub-module is used to create new Kafta topics dynamically in the Kafka Broker Cluster on new RIVA service creation. When a new RIVA service is created then three types of Kafka topics are automatically created with name convention RIVA_ID, RIVA_F_ID, and RIVA_A_ID on the Kafka Broker Cluster. Here ID is the unique identifier of the service. These topics are used to hold the actual video stream, IR, and anomalies detected by the IVA services. The Topic Manager sub-module permits the admin role to list all topics, check its configurations, and can also override the configurations if required. Each topic consists of partitions to which the video stream records are distributed. In general, the degree of parallelism and height throughput is proportional to the number of partitions in a topic. We develop a Partition Manager sub-module to automatically adjust the number of partitions in a topic according to video streams from video stream data sources and according to the stream consumption of the consumer group. To ensure height throughput and parallelism, a formula has been established for choosing the number of Partitions in a topic ‘T’. Let us consider, we want to achieve throughput ‘t’ for a producer ‘p’ and a consumer ‘c’ on a single partition. Then we need at least Max(t/p, t/c) partitions per topic.

For fault-tolerance, Apache Kafka provides Replication Protocol, and topic partitions can be replicated across Kafka Brokers. The Replication Protocol carries a parameter called Replication Factor (RF). An optimal value three is recommended for RF, but it must not exceed then the number of Broker Servers. In the Replication Manager sub-module, we develop APIs on the top of the Replication Protocol to manage the replication factor accordingly. The Cluster Configuration sub-module is responsible for holding and managing the cluster configuration parameters which are used by the producer and consumer, respectively. Similarly, a Cluster Health Manager sub-module is provided to allow the admin role to manage the health of the cluster.

#### 4.1.2. Video Stream Acquisition Service

**Figure 4:**
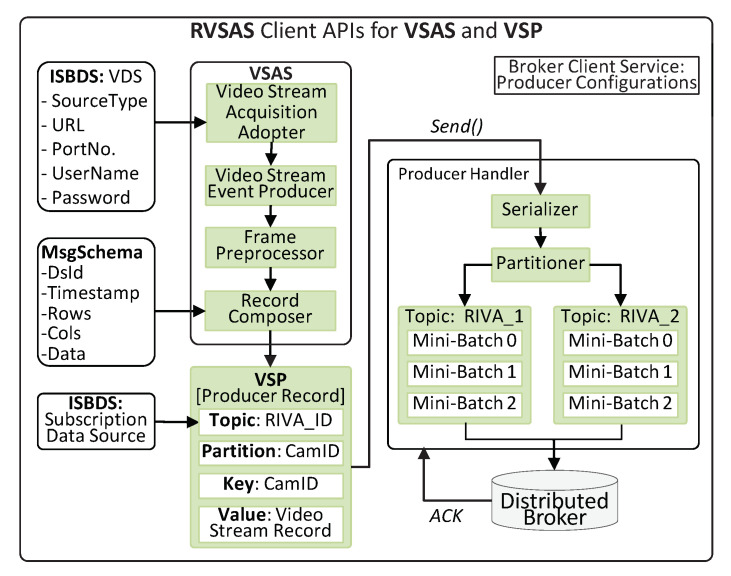
The internal logic and flow of the Video Stream Acquisition Service (VSAS) and Video Stream Producer (VSP).

VSAS are client APIs and can be configured on the Producer Cluster to acquire large-scale video stream. VSAS is composed of four sub-modules, i.e., Video Stream Acquisition Adaptor, Video Stream Event Producer, Frame Detector and Preprocessor, and Record Composed. The Video Stream Acquisition Adaptor provides interfaces for device independent video stream sources. If a particular video source is subscribed against a RIVA service, then the Stream Acquisition Service gets the configuration metadata from the Video Data Source DS in ISBDS and configure the source device for video streaming. Then the Video Stream Event Producer decodes the video stream, detects the frames, and forwards to the Frame Preprocessor for meta-data extraction and frame resizing. To communicate with the video stream source, a JavaScript Object Notation (JSON) object is defined. The contents of this object consist of five fields, i.e., data source id, number of columns and number of rows in a frame, the timestamp of the data origination at a data source and payload. This JSON object is known as Record, which is then forwarded to the Producer Handler, as illustrated in Figure 4.

#### 4.1.3. Video Stream Producer

The VSP are also client APIs and are deployed on the Producer Cluster. Its function is to receive the records from the VSAS module, serialize the records, form mini-batch and then send it to the Kafka Broker. If a video stream source is subscribed to RIVA service then the Producer Handler will rout the mini-batches to topic RIVA_1 in the Broker Cluster. Similarly, if subscribed to multiple RIVA services, then the stream will be routed to the respective topics, as shown in Figure 4. The Kafka Producer Record has a default message format and is composed of four fields, i.e., Topic Name, Partition Number, Key, and Value. The Topic Name is dynamically provided by the Subscription Data Source stored in ISBDS according to the video data source subscription. The Partition and Key fields are set to the video stream source ID (camera ID provided by the ISBDS Video Data Source meta-store). The input of the Value field is the Video Stream Record being composed and provided by the Record Composer. The Producer Recorded is then sent to the Serializer to convert the Producer Record to bytes array so that it can be sent over the network. The VSP collects the Producer Records and adds them to the batch of record, for sending to the Kafka Broker. We compress these mini-batches utilizing the snappy compression algorithm and then VSP sends these newly formed mini-batches to the Broker Server. We enable the ACK of the VSP to ensure the message delivery. Technically, the VSAS and VSP sub-modules are encapsulated in videoStreamEventProducer high-level API, as shown in Algorithm 1.

#### 4.1.4. Video Stream Consumer Services

**Figure 5:**
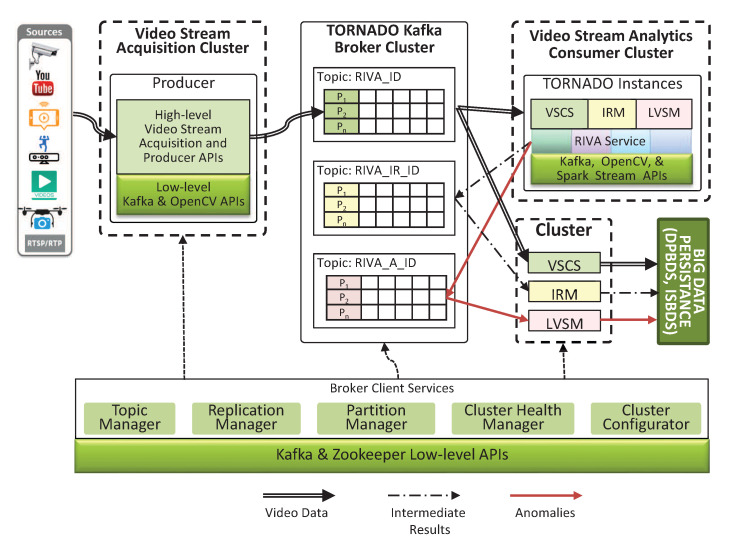
Video Stream Acquisition and Synchronization workflow.

The acquired video streams are now residing in the Kafka Broker in different topics in the form of mini-batches. To process these mini-batches of the video stream, we have different groups of computer clusters known as Video Stream Analytics Consumer Cluster. On each cluster, two types of client services are configured, i.e., RIVA services and VSCS. Each Video Stream Analytics Consumer Cluster has different domain-specific RIVA services whereas the VSCS are common for all. The VSCS assists a RIVA service to read the mini-batches of the video stream from the respective topic in the Kafka Broker for analytics, as shown in Figure 5.

**Algorithm 1:** videoStreamEventProducer(topicName, cameraID, cameraURL)

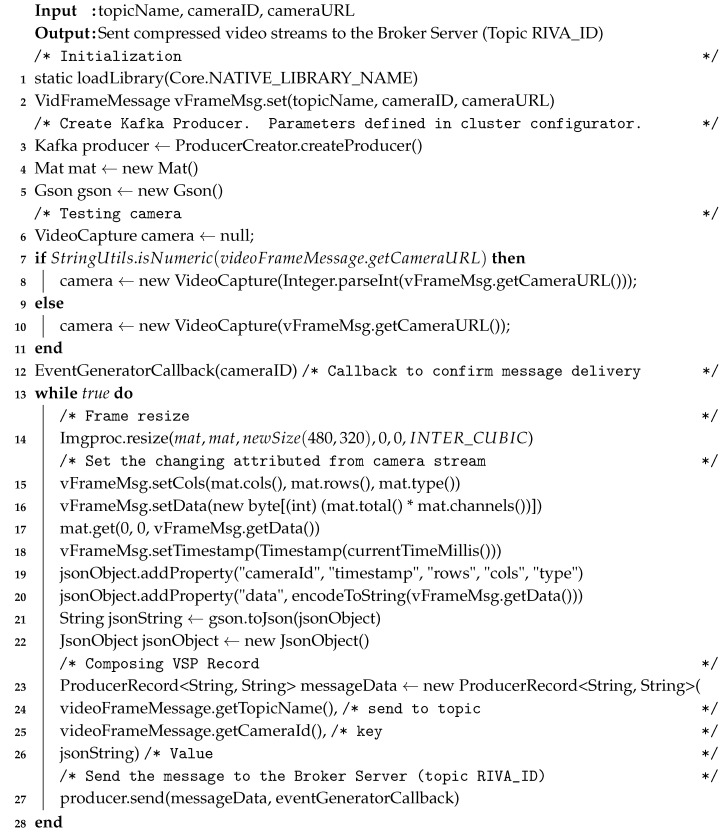



#### 4.1.5. Intermediate Results Manager

RIVA services generate IR and need to be persisted. Video analytics are always expensive and to avoid the recomputation, we persist the IR to ISBDS (IR Middleware). The IR demands proper management in a distributed environment. Thus in this context, we develop IR-Manager, which sends and gets the IR to and from the topic RIVA_IR_ID in the Kafka Broker Cluster. Similarly, this module is also responsible for reading IR from the respective topic and persists to the IR data store for reusability (such as mapping to video ontology, indexing for search) in order to avoid recompilation. The overall process is shown in Figure 5.

#### 4.1.6. Lifelong Video Stream Monitor

A domain-specific RIVA service processes the video stream for anomalies or abnormal activities. If any anomalies are detected, then the same is sent to the Kafka Broker topic (i.e., RIVA_A_ID) by using the LVSM instance. To generate the notification base response, LVSM follows standard observer-based implementation [33]. Based on this approach, the LVSM module reads the anomalies from the respective Kafka Broker topic, i.e., RIVA_A_ID and notifies the clients in near real-time and simultaneously persists to the ISBDS.

To understand the VSAS, IR-Manager, and LVSM, we create a template service (shown in Algorithm 2). This service is based on Apache Spark Streaming. The input to a RIVA service is the serviceID, and the subscribed cameraID. The video stream mini-batches are acquired from the topic RIVA_ID using the VSAS high-level abstraction and assigned to the videoDataset (instance of Spark dataset abstraction), as shown in step-4. RIVA operations (special or temporal analysis) are carried out on the videoData as per the developer logic and requirements. The RIVA output in the form of IR and anomalies are handed over to the IRM (step-5) and LVSM (step-6) APIs, respectively.

**Algorithm 2:** rivaServiceTemplate(serviceID, cameraID)

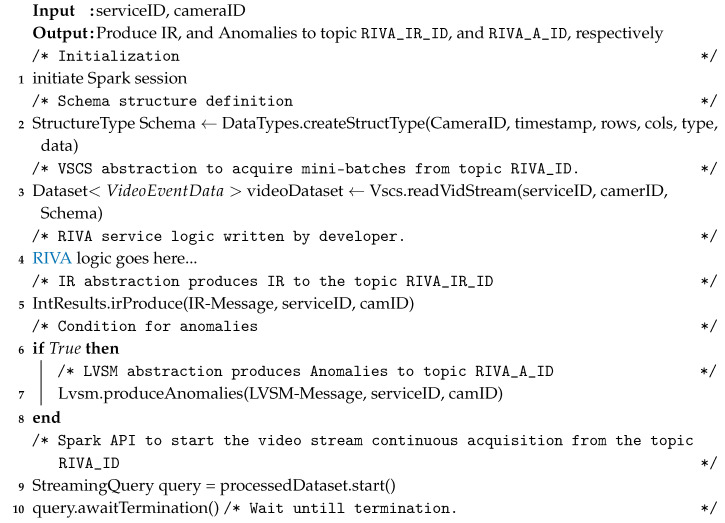



### 4.2. Big Data Persistence

The second component of the proposed framework is Big Data Persistence. The Big Data Persistence is the backbone of the TORNADO. The Big Data Persistence component is responsible for providing permanent and distributed big-data persistence to both the structured and unstructured data of the proposed platform. The Big Data Persistence provides two levels of abstraction on the acquired data, i.e., ISBDS and DPBDS, which are elaborated in the following subsections.

#### 4.2.1. Immediate Structured Big Data Store

**Figure 6:**
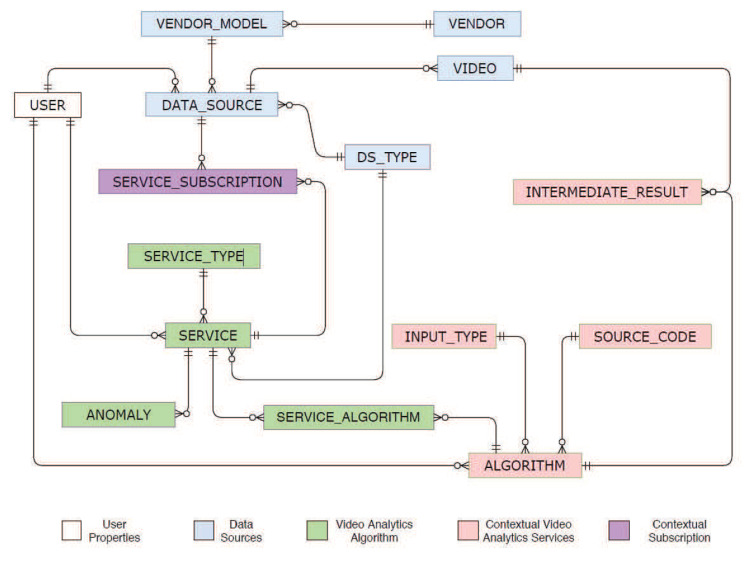
Immediate Structured Big Data Store model.

ISBDS is provided to manage large-scale structured data in the distributed environment. Because of the data-intensive operation and according to the requirements of the other layer, technologically, we deploy a distributed NoSQL big data storage known as Apache HBase. The NoSQL fits well according to the need and demands of the TORNADO, but it also has some limitations. It does not provide rich queries like SQL. Designing a complex system with HBase is a challenging task. Indexing can be challenging to design as a relational concept and is not implicit. An elegant solution to such issues exists in the form of Apache Phoenix [34]. Apache Phoenix is an SQL skin for HBase and enables OLTP operations best by combining the power of SQL and the flexibility and scalability of NoSQL. Thus, we exploit HBase as an NoSQL distributed data store and Apache Phoenix as a query execution engine. The ISBDS hosts five types of data, as shown in Figure 6. The detailed description of each type of data has been described subsequently.

##### 4.2.1.1. User Profile and Logs

TORNADO provides role-based access to users. User logs and the respective role information are maintained through the user catalog. All the sensitive user information is made secure by deploying an MD5 [35] encryption scheme.

##### 4.2.1.2. Data source Metastore

The proposed framework manages two types of video sources through Data Source metastore, i.e., video stream sources and video datasets. The former one can be subscribed to RIVA services, while the latter one is eligible for BIVA services. The meta-information of these sources, along with access rights, are managed through the Data Source metastore.

##### 4.2.1.3. Algorithm and Services Metastore

The IVA algorithms and services are managed through Video Analytics Algorithm and Service meta-store, respectively. The metadata of an IVA algorithm are managed through ALGORITHM metastore, and the data structure is composed of eight different data fields, i.e., AID, UID, algoType, algoName, input, output, requiredResources, sourceCodeAddress, and description. The metadata of the IVA service are managed through SERVICE metastore and encompasses attributes like SID, UID, dsTypeID, serviceTypeID, serviceName, and description. The relation between the service and algorithm metastore is managed through a lookup table called SERVICE_ALGORITHM.

##### 4.2.1.4. IR Middleware

**Figure 7:**
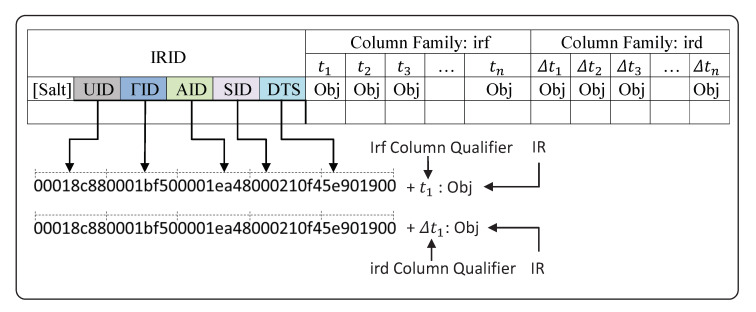
IR Middleware.

IVA algorithms and services generate heterogeneous types of results in the cloud and lead to the problem of big dimensionality. The TORNADO also demands a scale-out middleware to allow diverse types of IVA algorithms and services to communicate with each other. The produced IR also needs proper orchestration for reusability and to avoid recomputation. Against such diverse demands, we design a unified middleware called IR Middleware and it is the heart of the TORNADO framework. The IR Middleware is created on top of Hbase and is composed of two main parts, i.e., IRID, and IR Column-families, as shown in Figure 7.

**IRID Design:** The IR Middleware persists the extracted IR over time from videos. It has been optimized to support DateTime granularity-based queries of IR. This is accomplished through careful design of the row-key called IRID, as shown in Figure 7 in hex-encoded form. HBase store rows ordered by row-key, so the entire history for a single source is persisted as adjacent rows. Within the run of rows for a user, they are ordered by timestamp. The IRID are byte arrays comprised of the combination of <UID><ΓID><AID><SID><DTS>. The UID,ΓID,AID, and SID are monotonically increasing unique identifiers, which represent an instance of a user, video source, algorithms, and services, respectively. In the IRID, the DTS is a downsampled timestamp. When IRID is prefixed by an optional salt to distribute data across multiple regions better, then it is called SaltedIRID. In cryptography, a salt is random data that is utilized as an additional input to a one-way function to secure data [36]. Here our aim is not security but to generate a random hash value from an arbitrary argument. The timestamp is a Unix epoch four bytes encoded value in seconds. Rows are broken up into hour increments, reflected by the timestamp in each row. Thus, each timestamp will be downsampled to an hour value. This is to avoid stuffing too many IR in a single row as that would affect region distribution. Also, since HBase sorts on the row-key, data for the same user and time bucket, but either with different algorithms or services are grouped for efficient queries.

**Column Families:** The IR Middleware is composed of two types of column families, i.e., irf, and ird. The irf is utilized to persist the frame-level IR being extracted by an algorithm or service. The number of frames per second depends on the specifications of a video stream source. Let $F_{n}$ are the number of frames per second, then the total number of frames a bucket can accommodate will become Fn∗Seconds∗Minutes. The irf uses 12 bits rounded seconds as the column qualifier, as shown in Figure 7. The actual IR is persisted in the form of an object. Likewise, the ird column family is responsible for persisting spatiotemporal IR being extracted from a mini-batch by an algorithm or service. The irf uses a 24 bits column qualifier, which represents the time interval, i.e., first and last 12 bites are rounded seconds to identify the start and end of a mini-batch.

**Distributed IR Middleware:** By default, Hbase creates only one region for a table. Another split is created automatically when a table with one region gets large enough. Thus the deployment of IR Middleware will lead to the issues of hotspotting and load-balancing [37], which will straightaway affect the performance of TORNADO. Resultantly, the IR Middleware demands custom region creation and rows distribution across regions. To address these issues, let IR Middleware is split into $R_{\max}$ regions over Region Servers (RS). Each RS will get $R_{\max}/RS$ regions if $R_{\max}\geq RS$. When $R_{\max}<RS$, then $R_{\max}$ of the RS will be responsible for one *R*. Thus, some of the RS would not be used for IR Middleware. Additionally, against multiple regions, we utilize SaltedIRID and it is vital in the distribution of the rows across $R_{\max}$. For a given maximum value of the region range ($\lambda_{\max}^{IR}$), and a given number of $R_{\max}$, where $\lambda_{\max}^{IR}>R_{\max}$, the number of regions can be calculated as $\lambda_{\max}^{IR}/R_{\max}$.

**Figure 8:**
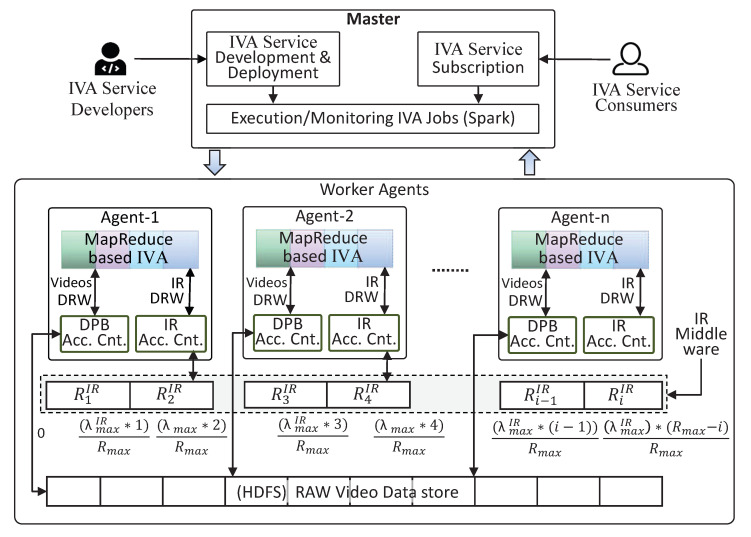
Working of Distributed Persistent Big Data Store (DPBDS) and Immediate Structure Big Data Store (ISBDS) Access Controllers in spark cluster environment.

In the interest of having $R_{\max}$ regions, we need to specify $R_{\max}-1$ split-points when creating the IR Middleware. For the proposed IR Middleware, the split-points can be $\left(\lambda_{\max}^{IR}*1\right)/R_{\max},\left(\lambda_{\max}^{IR}*2\right)/R_{\max},\ldots \left(\lambda_{\max}^{IR}*\left(i-1\right)\right)/R_{\max},\left(\lambda_{\max}^{IR}*\left(R_{\max}-i\right)\right)/R_{\max}$, as shown in Figure 8. With these split-points the i-th region, $i=1\ldots R_{\max}$, will handle the SaltedIRID in the range $\left(\lambda_{\max}^{IR}*\left(i-1\right)\right)/R_{\max},\left(\lambda_{\max}^{IR}*\left(R_{\max}-i\right)\right)/R_{\max}$. Given that each *R* will handle exactly $\lambda_{\max}^{IR}/R$ prefixes, it would be ideal if all regions are equally loaded. Aiming to achieve an even load on each *R*, we design the SaltedIRID as $(IRID\%R_{\max})+IRID$.

###### Subscription Metastore

Finally, users are allowed to subscribe video stream sources to a RIVA service. The subscription information are maintained through Subscription metastore.

###### ISBDS Access Controller

This module is responsible for providing read-write access to the underlying data securely according to the business logic of the TORNADO. This sub-module is composed of five sub-modules, i.e., Connection Configurator, Schema Handler, Create, Read, Update, and Delete (CRUD) Operator, Active and Passive Data Reader, and Writer.

Connection Configurator is used to maintain the configurations parameters such as cluster configuration, connection information, drivers, and security protocol parameters. When a request is made for data access, this sub-module is used to establish and maintain the connection session with the distributed data store. The basic schema structure of the ISBDS is maintained through Schema Manager, and the same can also be used for portability purposes.

CRUD is designed and provided for CRUD operations on data persisted in ISBDS. CRUD Operator is primarily designed to provide low-latency real-time interactive CRUD operations over the distributed storage. The instances of this sub-module are used by the distributed RIVA engines and by TORNADO Business Logic. It hosts pre-defined generalized queries for CRUD operations. Upon receiving the request for clients, CRUD Operator matches the request parameters with the available set of queries. In the case of a parameter match, the selected query is executed with a real-time response back to the client.

Furthermore, TORNADO is designed to support offline analytics over the bulk of videos while using distributed in-memory processing engines like Apache Spark [5]. The Passive Data Reader and Writer (PDRW) is provided to allow Apache Spark to load the bulk of data as RDDs (RDD is spark data structure [38]) and persist the same to the distributed tables as required. Execution of PDRW is a two-step process. First, the schema parameters are selected by the client application and then submit it to the PDRW. The PDRW dynamically generates query upon receiving the parameters. Then the query is executed over the NoSQL distributed data store, and the response is created and provided to the client program. The PDRW is provided to meet the demands of the offline analytics, and the query execution may take a longer time depending on the requested data size.

#### 4.2.2. Distributed Persistent Big Data Store

**Figure 9:**
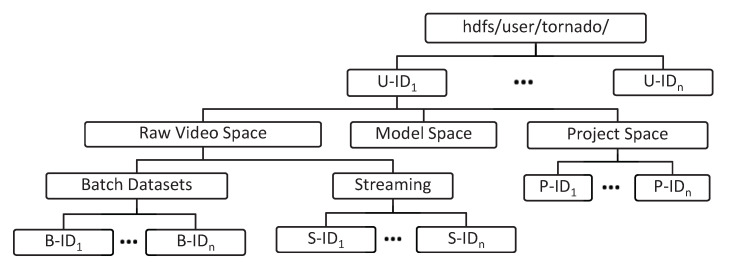
Realization of the hierarchical structure of user space in the Hadoop Distributed File System (HDFS).

The DPBDS component built on the top of HDFS and is responsible for providing permanent and distributed big-data storage. The data are stored and mapped systematically according to the business logic of the proposed system. The DPBDS component is designed to effectively and dynamically manage the data workload in the life cycle of IVA algorithms. Upon new registration with the TORNADO, a formal User Space is created on the top of HDFS. The User Space is managed through a proper hierarchical directory structure, and special read and write access is granted to the owner. All the directories are synchronized and mapped according to the user identification in the user profile logs. In HDFS, under each User Space three types of distributed directories are created, i.e., Raw Video Space, Model Space, and Project Space, as shown in Figure 9.

Raw Video Space is used for the management of the video data. The Raw Video Space is further divided into two types of videos. The first type is the batch video, which has been uploaded for batch analytics, where the second type is acquired and persisted from the video stream sources. The entire acquired stream is timestamped on persistence. The granularity level of raw streaming videos is maintained through video data sources. The IVA life cycle may need different models for training and testing purposes. The Model Space is provided to facilitate the users to manage the training and testing models according to the deployed IVA algorithm. Similarly, the developer can develop a new algorithm or service. The Project Space is provided to manage the source code of the respective developer.

To manage and operationalize the User Space, and the respective data in the DPBDS, we develop a DPBDS Access Controller module and it consists of three sub-modules. These sub-modules are HDFS Handler, Active DRW and Passive DRW. The former one is designed over low level APIs of HDFS with the aim of managing the file operations and permission control according to the business logic of TORNADO. The Active and Passive DRW are provided for real-time and offline read-write operations.

### 4.3. ISBDB Representation and Mapping

The ISBRM component works as a bridge between the TORNADO Business Logic component and the Big Data Persistence (BDP). This component is responsible for mapping the contextual data to the respective data stores according to the business logic of TORNADO. In Figure 3, the ISBDS module is shown as a cross cutting because it serves TORNADO Business Logic module. The ISBRM is composed of three modules, i.e., ISBDS Model Selector, Instance Mapper, and Data Validator.

As stated in Section 4.2, the structure and unstructured data of the proposed framework are maintained through BDP. The ISBDS data store is represented as an objected oriented abstraction called ISBDS Model, which encapsulates the attributes of the respective ISBDS data stores. The ISBDS Model is an implementation of Facade and Adopter Design Pattern [33]. The actual data mapping between the TORNADO Business Logic and ISBDS is possible because of the ISBDS Model Selector.

TORNADO communicates with the external word over web by following standard data exchange formats such as Extensible Markup Language (XML) [39] and JSON [40]. The Instance Mapper sub-module extracts the structured attributes from the input XML/JSON data as resources which are then mapped to the respective model instance. It identifies and maps the classes for the extracted resources and places the instances accordingly using a deep copy method [41]. Similarly, an XML/JSON object response is formed from the ISBDS dataset on retrieval and posted back to the client on request. The Data Validator sub-module validates the data according to the ISBDS schema and also ensures the compatibility with the defined constraints of the respective big-table in ISBDS.

### 4.4. TORNADO Business Logic

The BDP and RVSAS components are designed to support large-scale video data acquisition and domain-specific IVA in the cloud while exploiting an advanced IVA algorithm. The TORNADO Business Logic is a high-level abstraction on BDP and RVSAS and is provided to customize the data access according to the design philosophy of TORNADO. TORNADO Business Logic APIs utilization can be found in Appendix A.

In this context, the current version implements different modules in the TORNADO Business Logic components. The TORNADO Business Logic is subject to extendibility depending upon the future requirements. However, the current release implements and provide six types of modules, i.e., User Manager, Source Manager, IVA Algorithm and Service Manager, IVA Service Discovery and Subscription Manager, Ontology Data Manager, and Notification Manager.

**Figure 10:**
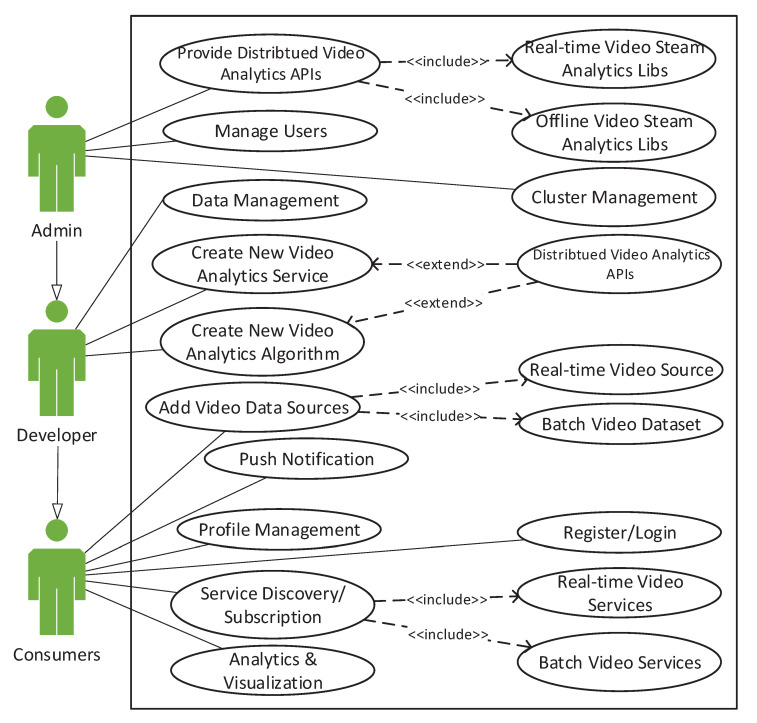
TORNADO user roles and use case diagram.

The User Manager module encapsulates all the user-related operations such as new user account creation, access role assignment, and session management. TORNADO provides role-based access to the user and currently provides three types of user accounts, i.e., admin, developer, and consumer. The user roles and access rights are shown in Figure 10. The user data access logic has been implemented in this module.

Thorough Data Source Manager module, the user can manage the data in a TORNADO deployed cloud. The users can manage three types of data sources, i.e., video stream sources, video datasets, and models. The IVA Algorithm and Service Manager modules are built to manage, develop, and deploy new IVA algorithms and services on the top of Spark. The former one is provided aaS, i.e., IVAAaaS to the developer, while the latter one is provided IVAaaS to the consumer. The developer can create and publish new IVA algorithms. These IVA algorithms are then made available as IVAAaaS to other developers for utilization. Once IVA services are created and published by the admin/developer, then TORNADO users are allowed to subscribe to video sources against the provided IVA services. The Ontology Manager allows the developer to get the IR for decision making. The Ontology Manager provides a secure way of getting the IR and maps it according to the video ontology deployed by the KCL. Similarly, Cluster Management are provided for administration purposes in order to allow the system administrator to monitor the health and functionality of the system.

**Figure 11:**
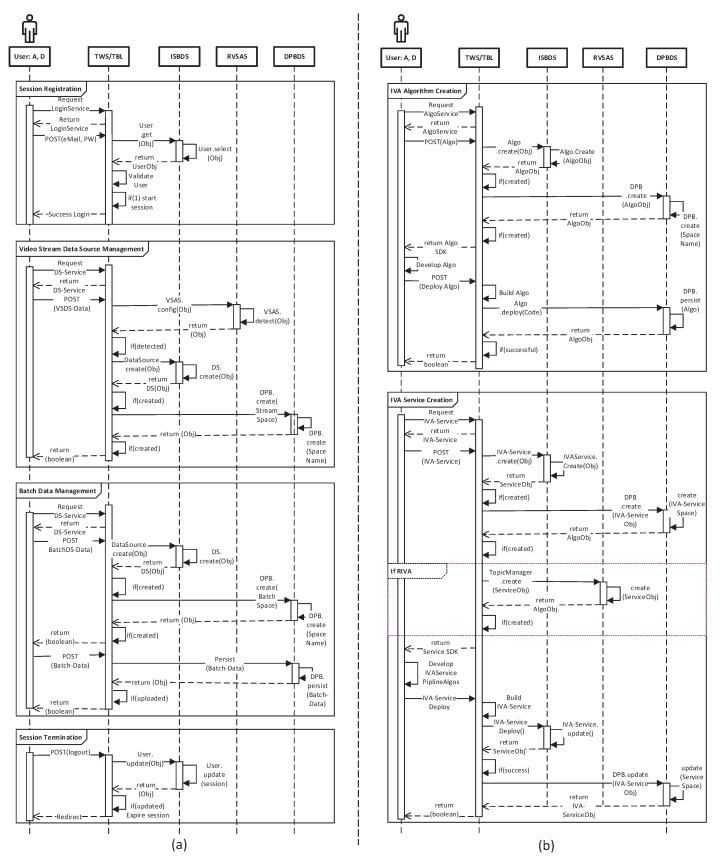
The sequence diagram for session creation, video stream data source, and batch data management are shown in (**a**). Similarly, the sequence diagram for the IVA algorithm and service creation is shown in (**b**). In the Actor lifeline, A and D represent Admin and Developer, respectively. These two roles are allowed to create a new video analytics algorithm and service.

Finally, to provide the functionality of the proposed TORNADO framework over the web, it incorporates top-notch functionality into simple unified role-based web services. The TORNADO Web Service Curation is built on the top of TORNADO Business Logic. The order and the intercommunication among different components of the proposed system are shown in Figure 11 through sequence diagrams.

## 5. Execution Scenarios

**Figure 12:**
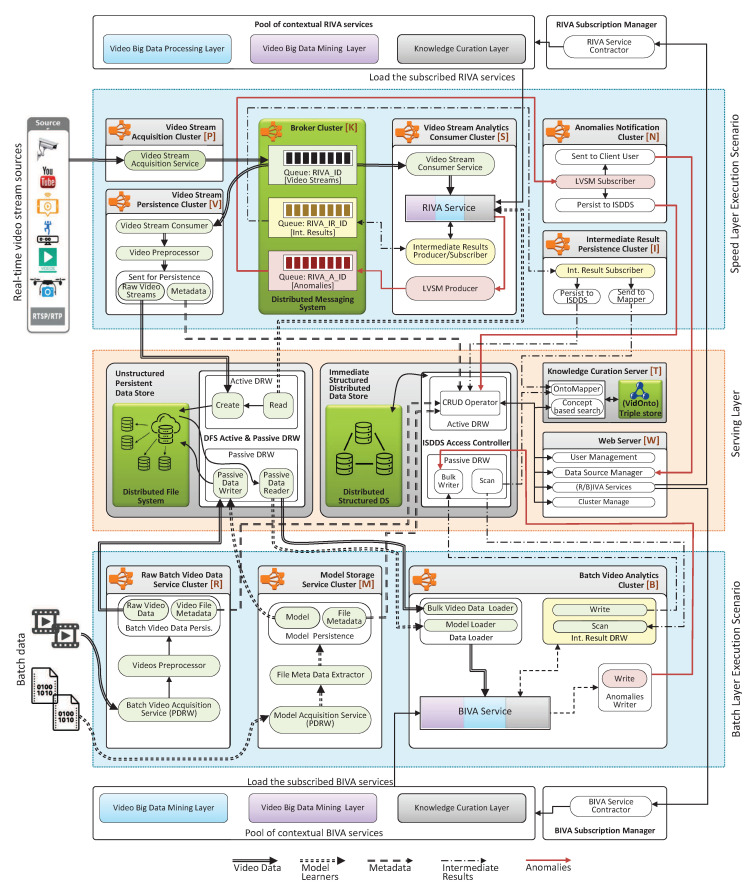
TORNADO execution scenarios.

TORNADO allows the participating layer to register distributed RIVA and BIVA services. The TORNADO follows the lambda architecture style [42] and the execution scenarios undergo two types of execution scenarios, i.e., Speed Layer Execution Scenario, and Batch Layer Execution Scenario. The data of both scenarios are managed through a common layer called BDP, also known as Serving Layer [42]. Figure 12 illustrates these execution scenarios, and the explanation is given in the following subsections.

### 5.1. Speed Layer Execution Scenario

TORNADO deploys a pool of RIVA services that are made available to the user for subscription. The IVA services are deployed by the supporting layers (VDPL, VDML, KCL). Once a video stream source is subscribed to a RIVA service in the pool of IVA services then the life cycle of Speed Layer Execution Scenario encompasses through different stages while using distinct TORNADO components. For the ease of understandability, these components are deployed on six types of computing clusters in the cloud, which are labeled explicitly as ‘P’, ‘V’, ‘K’, ‘S’, ‘N’, ‘I’, as shown in Figure 12.

The cluster ‘P’ hosts VSAS and provides interfaces to an external video stream source. On configuration, the video streams are loaded to the respective RIVA_ID, in the cluster ‘K’. The cluster ‘K’ deploys Kafka, where the acquired video streams, IR, and anomalies produced by LVSM are buffered. In this context, the cluster ‘K’ is composed of RIVA_ID, RIVA_IR_ID, and RIVA_A_ID, as described in Section 4.1. These topics are replicated to access cluster ‘K’ to ensure high throughput. The RF should be less than or equal to the number of workers in the cluster ‘K’ [43]. The mini-batches of video streams residing in the distributed broker’s topic RIVA_ID need to be persisted to the DPBDS and ISBDS data stores. For this purpose, the cluster ‘V’ deploys three types of TORNADO’s modules, i.e., VSCS, Video Processor and Persistence. The first module allows the cluster ‘V’ to read the video stream mini-batches from RIVA_ID topics in the cluster ‘K’. The cluster ‘V’ then processes, encode and extract the metadata from the consumed video data. Finally, the video stream persistence module saves the video data and the respective metadata to the DPBDS and ISBDS, respectively.

The cluster ‘S’ is responsible for processing video stream in near real-time while using RIVA services. Different stream processing engines, for example, Apache Spark Stream, can be used for the development of RIVA services. The cluster ‘S’ deploys four types of modules. The first module is VSCS and is used to consume the video streams from the RIVA_ID in the cluster ‘K’. The second type of module is the actual RIVA service that analyzes the video streams. The RIVA service is loaded according to the RIVA services subscription contract made by a user. The IR producer/subscriber is used to send and receive the IR according to the application logic to and from the topic IR in cluster ‘K’. The fourth type of module is the LVSM producer. A RIVA service instance deployed in the cluster ‘S’ should have some domain-specific goal and can produce anomalies if analyzed any. The TORNADO supports a real-time anomalies delivery system. The RIVA service sent the anomalies continuously to the LVSM producer and the LVSM producer to the respective anomalies topic RIVA_A_ID in the cluster ‘K’. The cluster group ‘I’ read the IR from the topic RIVA_A_ID in cluster ‘K’ continuously and sent it to the ISBDS’s IR Middleware for indexing. The final type of cluster in the active view is cluster ‘N’ and is known as Anomalies Notification Cluster. This cluster aims to read anomalies from the topic RIVA_A_ID in cluster ‘K’ and send the same to the ISBDS for persistence and also delivered in real-time to the video stream source owner in the form of alerts.

### 5.2. Batch Layer Execution Scenario

The TORNADO framework is also equipped with BIVA services. These are available aaS for batch video analytics. The batch video datasets are analyzed in an offline manner, where the execution time is proportional to the video dataset size and to the subscribed BIVA services. The Passive Execution life cycle undergoes three types of cluster, i.e., ‘R’, ‘M’, ‘B’. The cluster ‘R’ allows the user to upload a batch video dataset to the TORNADO cloud and configure three types of TORNADO libraries. The first type of service is Batch Video Acquisition Service, which is used to acquire a batch video dataset. Once uploaded to the node buffer, the batch dataset is processed by the activated Video Processor to extract the metadata from the batch videos. The batch video data and the extracted metadata are then persisted to the DPBDS and ISBDS, respectively. Similarly, the cluster ‘M’ works the same way as that of cluster ‘R’, but this one is responsible for model management.

In the batch video analytics, the supporting layers deploy BIVA services. This cluster loads the instance of BIVA services as per user contract and processes the videos in an offline manner. Once subscribed, this cluster loads the batch video dataset and model from the DPBDS. Similarly, the IR and anomalies are maintained in the ISBDS. The acquired video stream residing in the DPBDS is also illegible for offline analytics.

## 6. TORNADO Evaluation

TORNADO is a complex system and is composed of many components that are deployed over different types of clusters. Thus we evaluate various aspects of the proposed system. Before the evaluation, we explain the distributed cloud environment, which has been used for TORNADO evaluation.

### 6.1. Experimental Environment

**Figure 13:**
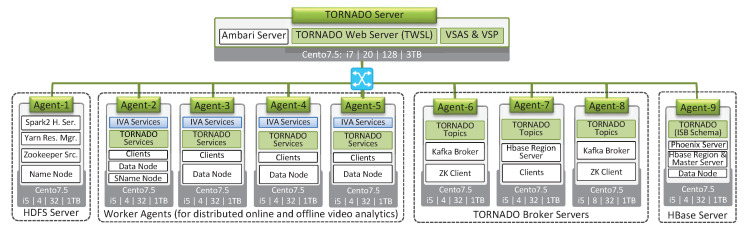
TORNADO Cluster.

For TORNADO, we set up an indoor distributed cloud environment called TORNADO Cluster while deploying Hortonworks Data Platform (HDP) version 3.1.0 [44]. The TORNADO Cluster consists of eleven nodes, as shown in Figure 13. Each node has five parameters. The top line parameter shows the operating system version being used. The second line shows the processor model, number of cores, size of RAM in GBs, and size of Hard-disk in GB, respectively. For the networking purpose, we use the ProSafe GSM7328S fully managed switches that deliver 24 and 48 ports of auto-sensing 1000 Mbps interfaces for high-density copper connectivity.

The TORNADO Cluster consists of five types of nodes, i.e., TORNADO Web Server, HDFS Server, Worker Agents, TORNADO Broker Servers, and HBase Server. The TORNADO Server hosts TORNADO Web Service, VSAS and video stream processing modules. This server also hosts Ambari Server [45]. The Agent-1 deploys HDFS Name Node [4], Zookeeper Server [46], Yarn Resource Manager [47] and Spark2 History Server [5]. Worker Agents is composed of four types of agents which deploy the TORNADO components (RVSAS, ISBDS, and DPBDS) and RIVA services (Face Detection and Action Recognition service). These nodes perform the actual near real-time analytics. We configure the TORNADO Broker Servers [32] on agent 6, 7, and 9 to buffer large-scale video stream, real-time IR, and LVSM alerts. The Agent-9 deploy TORNADO ISBDS schema on the top of HBase. Similarly, HBase Master has been configured on Agent-9 [37]. In Figure 13, Clients, and Data Node services are configured on some nodes. Data Node is the HDFS node where Clients means the instances of Spark, Yarn, Zookeeper, and HBase.

### 6.2. Services for Evaluation

We have developed two RIVA services, i.e., Face Detection and Action Recognition [29,48]. These applications are developed on top of Spark and the technical details of these services can be found in our previous publications, i.e., [29,48]. These two applications are available aaS. A user can expose real-time video stream sources for RIVA. On registration, these two services with TORNADO, three types of topics are automatically created by the Topic Manager (sub-module of BCS) as shown in Table 1. We set the RF to three (as we have three Broker server) and set the number of partitions to 140 per topic (to allow a maximum number of camera streams). Further, we set the value of different parameters in the Cluster Configurator sub-module, ISBDS, and DPBDS, as shown in Table 2.

### 6.3. Performance Evaluation and Scalability Testing of VSAS and VSP

**Figure 14:**
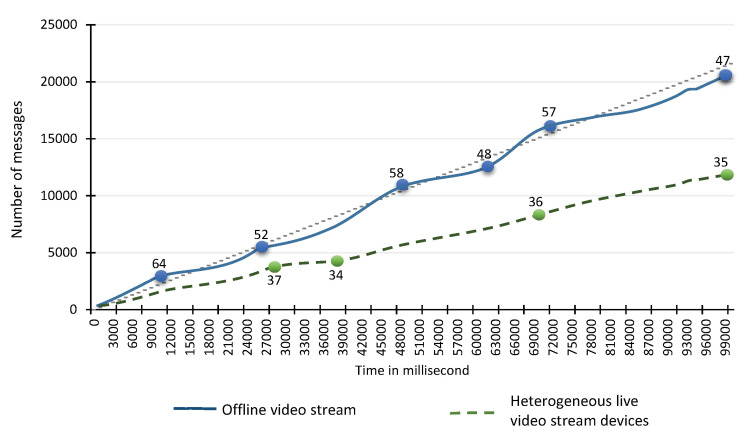
Performance testing of Video Stream Acquisition Service (VSAS) and Video Stream Producer (VSP).

As the VSAS module is device-independent, we register different types of heterogeneous devices and offline video stream sources. The heterogeneous video stream sources includes IP camera [49], depth camera [50], RTSP [51], and IPhone6s Plus [52]. By default, the frame rate of the first three data sources is 30, and the last one is 60 frames per second. In the case of an offline video stream source, a video file residing on HDD (WDC WD10EZEX) has also been configured with VSAS sub-component. The VSAS set the resolution of the acquired frame to 480 × 320 pixels. Resultantly, the size of each acquired frame became 614.495 KB. The VSAS converts the acquired frame to a formal message at the rate of 6 MS. The VSAS then forwards the message to VSP. The VSP compresses the size of the message to 140.538 KB on average and forward to the TORNADO Broker Server at the rate of 12 MS. These two modules are configured on TORNADO Server to acquire and send the stream to the TORNADO Broker Servers (Agent-6, Agent-7, and Agent-8). The results of the performance testing are shown in Figure 14. From the results, it is quite clear that, on average, we can acquire 34 and 54 frames per second from the heterogeneous video stream sources and offline video stream sources, respectively. The achieved rate is 36% and 116% percent higher than the preferred, i.e., 25 frames per second for RIVA analytics.

**Figure 15:**
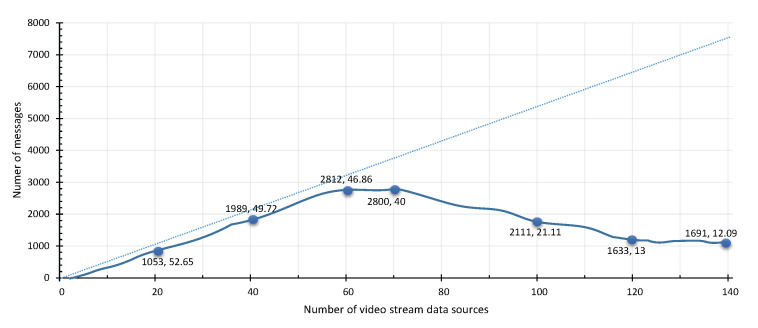
Stress testing of scalability of VSAS and VSP.

To evaluate the ability of TORNADO to scale per system with the increase in video stream sources. We increase the number of video stream sources on the TORNADO Server from 5 to 140. This test case stresses the VSAS and VSP sub-components with an average of 54 messages per second per video stream source. The respective results are shown in Figure 15, which shows that the VSAS and VSP module can acquire and produce a stream from 70 devices successfully. It can acquire and produce 40.42 messages per video stream source. As we add more devices, the performance degrades. However, we recommend attaching up to 70 video stream sources per system. As we are using a distributed messaging system, i.e., Kafka, which can scale out easily by adding more producers and broker servers.

**Figure 16:**
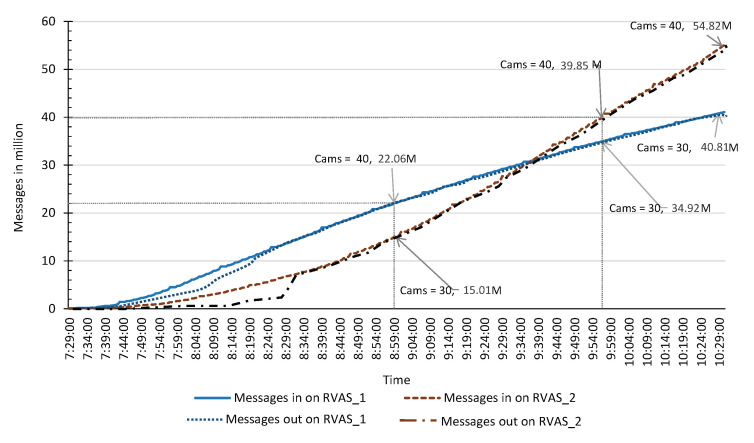
VSAS and VSP performance in production environment.

The TORNADO stream acquisition component is then tested in a production environment while subscribing to the RIVA services. Initially, we register and subscribe 30 and 40 cameras with the Face Detection and Action Recognition services, respectively. The VSAS and VSP successfully acquire and sent the messages to topic RIVA_1 and RIVA_1 on TORNADO Broker servers. Furthermore, after 1.5 h, we unsubscribe 10 cameras from Face Detection service and re-subscribe the same to Action Recognition service. Up to three hours, we receive 95.63 million messages on the TORNADO Broker servers as shown in Figure 16. On average, we receive 2952.16 messages per second.

### 6.4. Performance Evaluation of Video Stream Consumer Service

**Figure 17:**
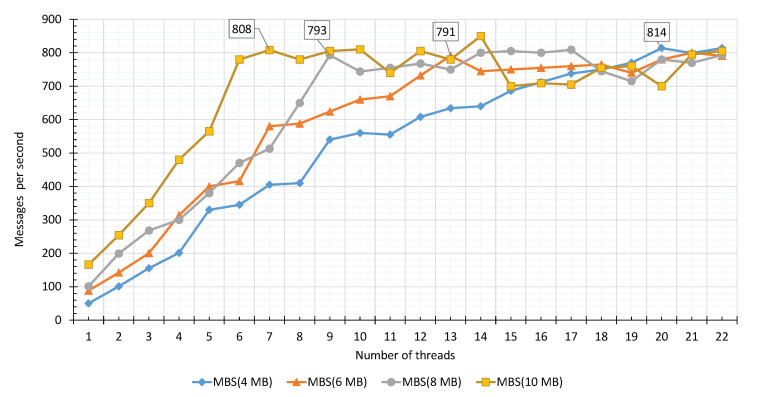
Performance testing of VSCS.

In this section, we evaluate the performance of VSCS module. VSCS acquire the video streams from the TORNADO Broker server in the form of mini-batches for analytics. The $\Delta t_{i}$ size is significant in the context of IVA service (especially in temporal IVA). The size of the mini-batch is dependent on MAX_REQUEST_SIZE_CONF variable. For the evaluation, we set up four different cases, i.e., Case-1, Case-2, Case-3, and Case-4, as shown in Table 3, while setting the mini-batch size to 4, 6, 8, and to 10 MB, respectively. With synchronous replication, a single thread on a single worker node achieves 50, 88, 101, and 166 messages per second on average. In each case, we initiate 22 threads to receive mini-batches from the TORNADO Broker servers. In Case-1, we can achieve the best performance in 20 threads while receiving 814 messages per second. Similarly, in Case-2, 3, and 4, the optimal performance was achieved in 13, 9, and 7 Threads, as shown in Table 3. Adding more threads per system does not increase performance, as shown in Figure 17. The effect of the VSCS in the production environment is shown in Figure 16 where the messages out on RIVA_1 and RIVA_1 are almost overlapping with that of the receiving. For message consumption, we use worker agents.

### 6.5. Lifelong Video Stream Monitor

**Figure 18:**
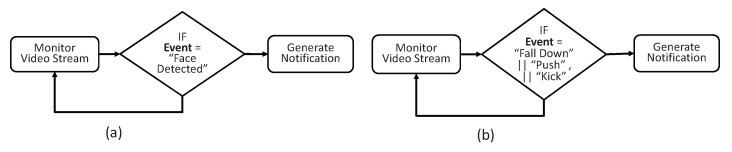
Anomaly detection rules. (**a**) show rules for event type “Face Detection”, i.e., when a face in a video stream is detected, then a notification is generated. Similarly, (**b**) show anomalies detection rules for abnormal events, i.e., when an abnormal event like Fall Down, Push, or Kick is detected in a video stream, then a notification is generated accordingly.

The LVSM component is responsible for notifying the TORNAOD’s user in near real-time after the detection of some abnormal activity in the video stream by domain-specific RIVA service. We subscribe Camera-1 and Camera-2 to Face Detection and Action Recognition services, respectively. The rules of these anomalies are shown in Figure 18.

**Figure 19:**
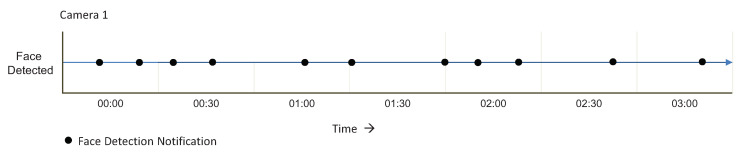
Camera 2 video stream monitor against the service Face Detection for three hours.

The monitored lifelong of Camera-2 is shown in Figure 19. In the figure, we can see that faces have been detected in near real-time, and the respective notifications are generated.

**Figure 20:**
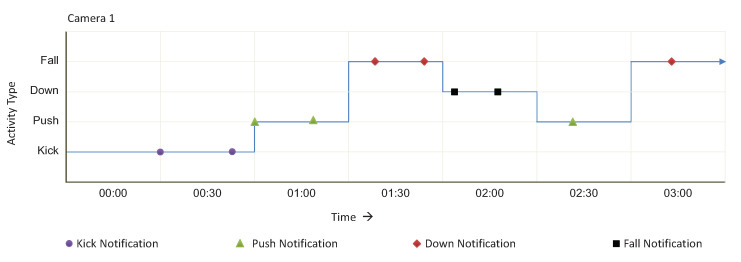
Camera 2 video stream monitor against the service Activity Recognition for three hours.

Similarly, the Action Recognition service monitors the lifelong video stream of Camera-1 according to rules given in Figure 18. In Figure 20, we can see that various types of activities have been generated with time.

**Figure 21:**
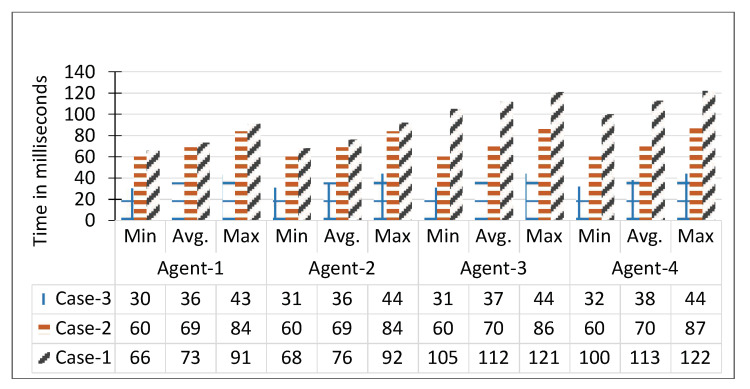
Notification delay performance evaluation.

**Figure 22:**
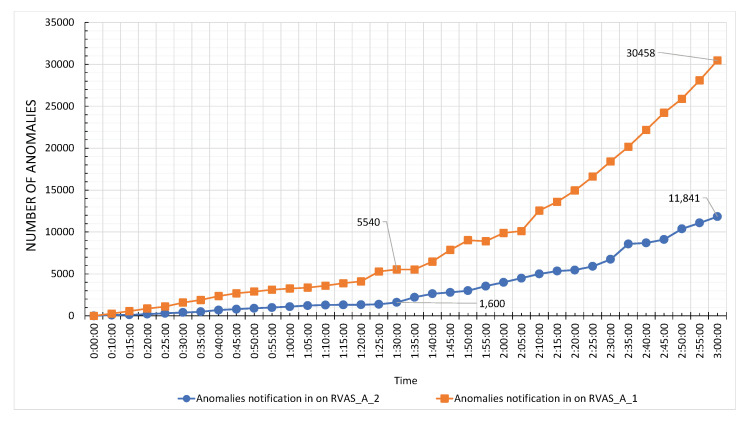
Total number of anomalies produced by Face detection and Activity recognition.

The performance of LVSM is based on its timely generation of notification for the real-time domain-specific video analytics service. For the LVSM evaluation, we introduce three types of delay cases, i.e., Case-1, Case-2, and Case-3. The life cycle of Case-1 considers the total time from frame acquisition to processing and then the notification. The delay of the notification, in this case, is proportional to the execution time of the RIVA service. In Case-2, we exclude the execution time, whereas in Case-3, only the delay between the occurrence of anomaly and publication of notification is evaluated. The LVSM services are configured on the agents of the work. For this evaluation, Camera-1 was subscribed to Face Detection service and Camera-2 to Action Recognition. In Case-1 the notification delay, on average for Face Detection and Action Recognition, 73 and 113 milliseconds were recorded. In Case-2, the average notification delay is 69, and 70 milliseconds was achieved. Similarly, in Case-3, we get 36 and 38 milliseconds on average. The delay comparison of LVSM is shown in Figure 21. From these statistics, it is clear that LVSM publishes notification with the highest efficiency. During the video stream processing by worker nodes, the Face Detection and Action Recognition generates 30,458 and 11,841 notifications and sends the same to the topic RIVA_A_1 and RIVA_A_2, respectively, as shown in Figure 22.

### 6.6. Performance Evaluation of Intermediate Results Manager

**Figure 23:**
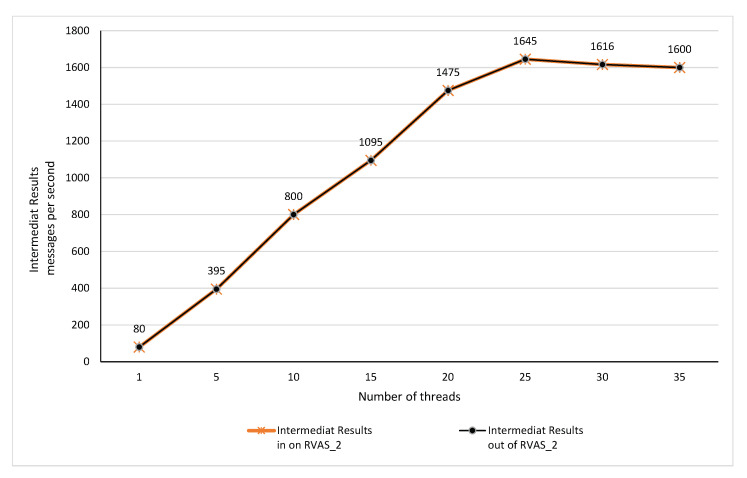
Performance evaluation of Intermediate Results Manager.

**Figure 24:**
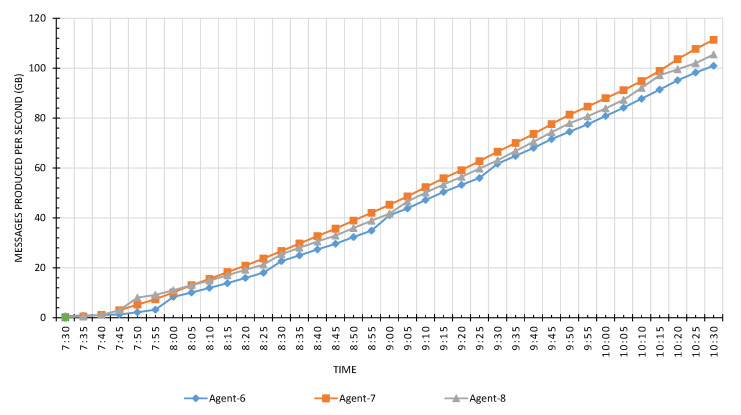
Total data consumed by the Broker Servers.

As the TORNADO is supposed to provide support for the IVA life cycle and to manage the IR. Once the features are extracted from the mini-batches of videos, the same is then produced for the respective topic. Face Detection produces bounding boxes, whereas Action Recognition services generate low-level features, i.e., VLBP [48]. These are sent to the RIVA_IR_1, and RIVA_IR_1, respectively on the TORNADO Broker server. On average, each mini-batch of the video stream generates a feature vector of 100 KB against the VLPB algorithm, which becomes 40 KB after compression while using snappy compression. A single node using a single thread can process 80 messages per second. When using four consumers and four producers with 25 threads each, we get up to 1645 messages per second, as shown in Figure 23. During the experimental analysis, the Action Recognition service generates 0.4 million VLPB features and sends it to the respective topic TORNADO Broker server successfully. Finally, Figure 24 also shows the total data consumed (including video streams, anomalies, and IR) by the Broker servers. As we are using the RF three; thus, the data are accurately replicated to the three broker servers which ensure the fault-tolerance.

### 6.7. IR Middleware Evaluation

In this section, we evaluate the performance of IR Middleware. We utilize the IR-Manager to read the IR from RIVA_IR_1 and RIVA_IR_2, and then call the IR Access Controller to persist the same to the IR Middleware. To study the effect of the number of regions on the parallelism, based on the discussion in Section 4.2.1.4, we pre-split the IR Middleware by setting $R_{\max}=12$, and $\lambda_{\max}^{IR}$ to hex-encoded values for the regions in the range ${}^{\prime }00000000^{\prime }$ - ${}^{\prime }FFFFFFFF^{\prime }$. These splits are distributed over four RS. The RS are configured on Agent-2, 3, 4, and 5. Thus each RS servicing three regions of the IR Middleware.

**Figure 25:**
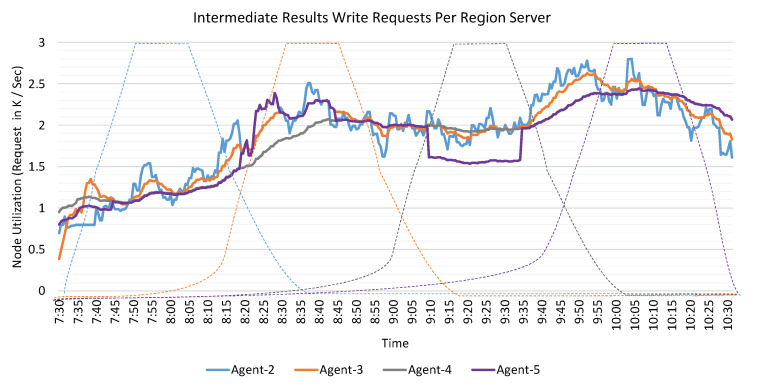
Performance evaluation of Intermediate Results (IR) Middleware.

The write performance of the IR Middleware is shown in Figure 25 while exploiting the proposed SaltedIRID. It is clear from Figure 25 that the write operations are almost distributed equally over the designed IR Middleware. In Figure 25, the dotted lines show that the utilization of IRID will persist the IR to the Agent-2 first, then to 3, and so on. Through the IRID, it will lead to the issue of hotspotting.

### 6.8. Distributed Persistent Big Data Store

**Figure 26:**
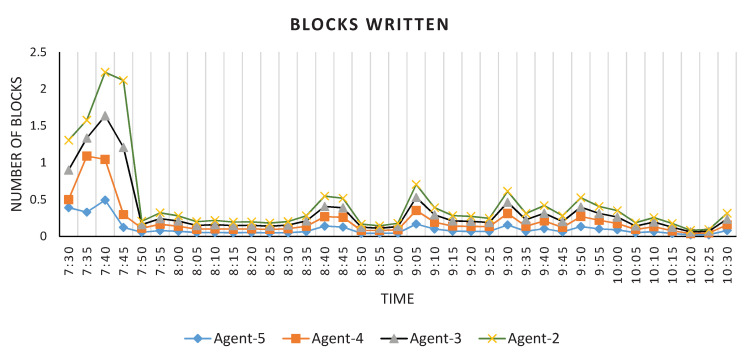
Performance of Distributed Persistent Big Data Store (Active Data Writer).

**Figure 27:**
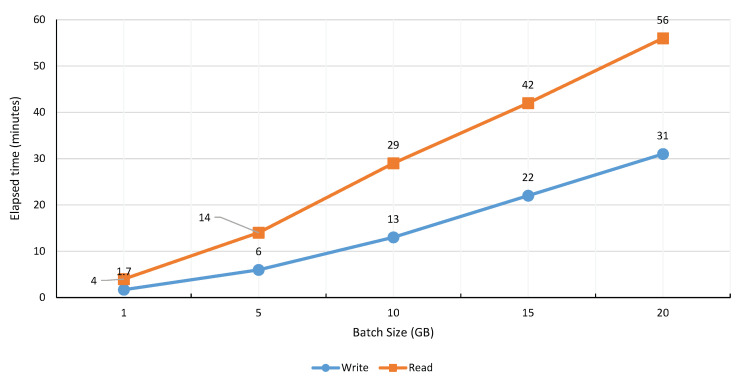
Performance evaluation of Passive Data Reader and Writer.

To evaluate the DPBDS performance over HDFS, we have performed experimentation both on Active and Passive Data Reader and Writer. The HDFS instances are configured on the worker agents (Data Nodes) and HDFS Server (Name Node) as shown in Figure 13. Likewise, the Active and Passive Data Reader and Writer are configured on the worker agents and TORNADO Server. The Active Data Writer consumes the video stream from the topic RIVA_ID (TORNADO Broker server) and persists the video stream to the HDFS. The performance result of the Active Data Writer, i.e., blocks that are written to the data node, is shown in Figure 26. From the results, it is clear that the Active Data Writer ensures the data locality and proper data distribution. Similarly, we evaluate the performances of Passive Data Reader and Writer operations (illustrated in Figure 27 over batch video data). These operations have been executed for five different batch video sizes, i.e., 1, 5, 10, 15 and 20 GB. The results show that the write operation is faster than the read operation. The time difference for both read and write is proportional to the volume of batch video data.

## 7. Discussion

This section discusses the features of the proposed TORNADO that are related to state-of-the-art CVAS. Furthermore, it also highlights features of the proposed system that leverage it from existing systems. The comparison of the proposed system with commercial and scholarly work is shown in Table 4.

### 7.1. Common Features with Existing Systems

Some of the features of the proposed system are similar to the existing commercial and scholarly systems. Commercial CVAS like Google Vision [15], and IBM CVAS allows the users to subscribe to the batch videos to the provided BIVA services. At maximum, the user can utilize their domain-specific models. The Azure Video Analytics [53], Citilog [54], CheckVideo [13], and IntelliVision [14] support real-time IVA, and one can subscribe to the video stream to existing IVA services. The proposed system shares some features of video data acquisition of the CVAS proposed in [10,11,22,27].

### 7.2. Differences with Existing Systems

The principal dissimilarities between the proposed CVAS and the state of the art solutions are listed below.

- Unlike existing CVAS, the TORNADO framework is intended to provide a service-oriented echo-system while utilizing open-source big data technologies to facilitate IVA developers and scientists. Users can develop and deploy new IVA algorithms under IVAAaaS. The IVA algorithms can then be pipelined to create a domain-specific IVA service. The users can then subscribe to video sources to the available IVA services.
- In the context of IVA, the proposed system provides higher-level abstractions on the low-level APIs on top of big data solutions, which assist users to focus more on the IVA solution.
- The proposed system is equipped with IR-Manager and LVSM to maintain and manage the IR and anomalies.
- Unlike existing solutions, the proposed framework is facilitated with scale-out IR Middleware, which addresses the issue of big dimensionality. The IR Middleware allow the integration of diverse types of IVA services with TORNADO.
- The proposed system is based on the lambda architecture and can easily be extended to the fog based solution, i.e., RIVA services can be deployed near to the video stream data sources and BIVA service on the cloud resources.

## 8. Conclusions

In this paper, we presented the TORNADO framework, which focuses on curating large-scale video data in the cloud environment while deploying IVA algorithms and services under an aaS model. TORNADO is a pluggable and scale-out framework, which is designed to bridge the gap between IVA algorithms and service developers. It provides high-level abstractions on top of big data stacks to allow researchers/developers to focus more on the IVA services in the cloud. TORNADO efficiently manages the IR during the IVA life cycle. Furthermore, IVA algorithms can easily be pipelined to create a domain-specific IVA service. The data source-independent implementation of TORNADO makes it more scalable and IoT compatible. TORNADO is also facilitated with LVSM, which monitors the video stream against the rules defined in the IVA service by domain experts. Distributed Big Data Persistence is provided to support a large volume of raw video data, models, structured data, and the IR, which enables the TORNADO to support data-driven knowledge generation, descriptive and predictive analysis, and visualization.

The TORNADO framework performance, accuracy, and scalability have been successfully evaluated against the demands of the TORNADO for real-time and offline IVA. From the results, it is clear that the proposed framework performs efficiently and effectively in the production environment.

The current version has been validated against RIVA services. In the future, we will evaluate and optimize the IR Middleware against tree data structures, and will provide more IVA services, i.e., offline video analytics, and deep-learning-based approaches. In the future, we are also investigating to optimize the TORNADO against in-memory computing engines like Apache Spark.

## Figures and Tables

**Table 1:** Topics for Face Detection and Action Recognition services.

Topic Name	Description	RF	Partitions
RIVA_1	Consumes the stream, subscribed to Face Detection service.	3	140
RIVA_IR_1	Consumes the IR of Face Detection service.	3	140
RIVA_A_1	Consumes the anomalies of service Face Detection.	3	140
RIVA_2	Consumes the streams, subscribed to Action Recognition.	3	140
RIVA_IR_2	Consumes the IR of Action Recognition service.	3	140
RIVA_A_2	Consumes the Anomalies of Action Recognition service.	3	140

**Table 2:** Real-time video stream and acquisition component parameter settings.

	Variable	Value
Kafka	ACKS_CONFIG	All
	BATCH_SIZE_CONFIG	20,971,520 Bytes
	COMPRESSION_TYPE_CONFIG	Snappy
	MAX_REQUEST_SIZE_CONFIG	2,097,152 Bytes
	LINGER_MS_CONFIG	5
	AUTO_COMMIT_INTERVAL_MS_CONFIG	1000
HDFS	Block replication	3
	HDFS Block Size	64 MB
	Java heap size	1 GB
HBase/Phoenix	phoenix.query.timeoutMs	1,800,000
	hbase.regionserver.lease.period	1,200,000
	hbase.rpc.timeout	1,200,000

**Table 3:** Performance evaluation of Video Stream Consumer Services (VSCS).

	MBS Size	Avg. Msgs/Sec	Max thread (Optimal)	Avg. Msgs/Sec
Case-1	4 MB	50	20	814
Case-2	6 MB	88	13	791
Case-3	8 MB	101	9	744
Case-4	10 MB	166	7	808

**Table 4:** Feature-wise comparison with state-of-the-art Cloud-based Video Analytics System (CVAS).

	Role-Based Secure Access		Video Data Acquisition				Data Maintenance & Management		IVA aaS				Processing Engines Support		
	Developer	Consumer	Video Stream	Batch Video	Model	API	IR Midd.W.	Anomalies	RIVA-aaaS	RIVA-aaS	BIVA-aaaS	BIVA-aaS	Spark Stream	SparkMR	HadoopMR
**Google vision [15]**	✓	✓	✗	✓	✓	✗	✗	✗	✗	✗	✗	✗	✗	✗	✗
**Azure CVAS [53]**	✓	✓	✓	✓	✓	✗	✗	-	✗	✓	✗	✗	-	-	-
**IBM CVAS**	✓	✓	✗	✓	✓	✗	✗	✗	✗	✗	✗	✓	-	-	-
**Citilog [54]**	✗	✓	✓	✗	✗	✗	✗	✓	✗	✗	✗	✗	✗	✗	✗
**CheckVideo** [13]	✗	✓	✓	✗	✗	✗	✗	✗	✗	✗	✗	✗	✗	✗	✗
**IntelliVision** [14]	✗	✓	✓	✗	✗	✗	✗	✗	✗	✗	✗	✗	✗	✗	✗
**Liu, X. et al. [10]**	✗	✗	✗	✓	✗	✗	✗	✗	✗	✗	✗	✗	✗	✗	✓
**Zhang, W. et al. [22]**	✗	✗	✓	✓	✗	✗	✗	✗	✗	✗	✗	✗	✗	✗	✓
**Zhang, W. et al. [11]**	✗	✗	✓	✓	✗	✗	✗	✗	✗	✓	✓	✗	✗	✗	✓
**Ganesh, A. et al. ** **[26,27]**	✗	✗	✓	✗	✗	✗	✗	✗	✓	✓	✗	✗	-	-	-
**TORNADO**	✓	✓	✓	✓	✓	✓	✓	✓	✓	✓	✓	✓	✓	✓	✓

## Abbreviations

The following abbreviations are used in this manuscript:

aaS	as-a-Service
BCS	Broker Client Services
BDP	Big Data Persistence
BIVA	Batch IVA
C2C	Customer-to-Customer
CRUD	Create, Read, Update, and Delete
CVAS	Cloud-based Video Analytics System
DPBDS	Distributed Persistent Big Data Storage
DRW	Data Reader and Writer
HDFS	Hadoop Distributed File System
HDP	Hortonworks Data Platform
IoT	Internet of things
IR	Intermediate Results
IR-Manager	Intermediate Results Manager
ISBDS	Immediate Structured Big Data Store
ISBRM	ISBDS Representation and Mapping
IVA	Intelligent Video Analytics
IVAAaaS	IVA-Algorithm-as-a-Service
IVAaaS	IVA-as-a-Service
JSON	JavaScript Object Notation
KCL	Knowledge Curation Layer
LVSM	Lifelong Video Stream Monitor
PDRW	Passive Data Reader and Writer
RF	Replication Factor
RIVA	Real-time IVA
RS	Region Servers
RVSAS	Real-time Video Stream Acquisition and Synchronization
VDML	Video Data Mining Layer
VDPL	Video Data Processing Layer
VSAS	Video Stream Acquisition Service
VSCS	Video Stream Consumer Services
VSP	Video Stream Producer
XML	Extensible Markup Language

## Appendix A. TORNADO Business Logic APIs Utilization

The TORNADO Business Logic allows the user to communicate with TORNADO. The utilization of the TORNADO Business Logic and the communication among different components are shown in the sequence diagrams in Figure 11. Here in the following subsection, we demonstrate some real examples, i.e., how to interact with TORNADO and how to utilize the high-level APIs of the TORNADO Business Logic. Furthermore, TORNADO framework can be download from GitHub (https://github.com/angry-bit/TORNADO).

### Appendix A.1. User Manager

*//Set new user attributes* *//userRoles: 0 = Admin, 1 = Developer, 2 = Consumer* User user = **new** User (); user.setFirstName (“firstName”).setLastName (“lastName”) .setUserName (“userName”).setEmail (“email”) .setUserRole (userRole).setPassword (“password”); *//Register a new user.* UserDAO userDAO = **new** UserDAO (); userDAO.create (user); *//User Login, if already registered.* userDAO.login (“email”, “password”);

### Appendix A.2. Video Stream Data Source Manager

*//dsType: 0 = Video stream source, 1 = Batch data source, 2 = ML Model* DataSource dataSource = **new** DataSource (); dataSource.setUserId (“userId”) .setCameraUserName (“cameraUserName”) .setCameraPassword (“cameraPassword”) .setCameraLink (“cameraLink”) .setDescription (“Camera Description if requried”); DataSourceDAO dataSourceDAO = **new** DataSourceDAO (); dataSourceDAO.create (dataSource, dsType); *//Access a single camera with a given ID* dataSourceDAO.get (ds_id, dsType); *//List all camera of a user.* dataSourceDAO.getAll (user_id, dsType);

### Appendix A.3. Batch Data Source Management

*//First create a dataset directory* dataSource.setDsName (dsName) .setUserId (userId) .setDescription (description) .setdsType (dsType); dataSourceDAO.create (dataSource); *//Upload Videos* DataSourceDAO dataSourceDAO = **new** DataSourceDAO (); dataSourceDAO.upload (dsName, sourcePath); *//Access model* dataSourceDAO.get (videoDatasetName);

### Appendix A.4. Model Management

*//Machine Learning model management* dataSource.setDsName (modelName) .setDescription (description) .setdsType (dsType); *//Upload Model* dataSourceDAO.create (dataSource); dataSourceDAO.upload (modelName, sourcePath); *//Access model* dataSourceDAO.get (modelName);

### Appendix A.5. RIVA Service Creation and Registration

Like RIVA service, BIVA services can be created, and registered with the TORNADO

*//serviceType: 0 = RIVA service, 1 = BIVA Service* Service service = **new** Service (); service.setServiceName (“serviceName”).setServiceType (serviceType) .setUserId (userId).setDescription (“Service description”); ServiceDAO serviceDAO = **new** ServiceDAO (); serviceDAO.create (service); *//Create RIVA service as per algorithm 2.* *//Convert to Jars and Upload to the TORNADO.* serviceDAO.registerService (serviceName, pathToJars);

### Appendix A.6. Service Discovery and Subscription

*//List RIVA services* ServiceDAO.getAll (serviceType); *//Set service subscription properties* ServiceSubscription serviceSubscription = **new** ServiceSubscription (); serviceSubscription.setServiceId (serviceSubscriptionId) .serviceSubscription.setDsId (dsId) .setSubscriptionStartDate (subscriptionStartDate) .setSubscriptionStopDate (subscriptionStopDate); *//Subscribe a RIVA service.* ServiceSubscriptionDAO.create (serviceSubscription);

### Appendix A.7. Ontology Data Manager

*//Explore Intermediate Results of an RIVA video annotation serviec* *//Set the basic attributes* IntermediateResult intermediateResult = **new** IntermediateResult (); intermediateResult.setdSID (videoStreamSourceID) .setSID (videoAnalyticsServiceID).setUID (userID) .setStartTimestamp (startTimestamp).setStartTimestamp (endTimestamp) IntermediateResultDAO.getIR (IntermediateResult);

### Appendix A.8. Anomalies

*//Exploring Anomalies* Anomaly anomaly = **new** Anomaly (); anomaly.setDsId (dsId).setServiceId (serviceId).setUID (userID) .setStartTimestamp (Timestamp).setEndTimestamp (Timestamp); AnomalyDAO anomalyDAO = **new** AnomalyDAO (); anomalyDAO.get (anomaly);
